# Do new trainees pose a threat to postoperative complications after hip fracture surgeries? Retrospective cohort of 1045 patients over a decade at a university hospital

**DOI:** 10.1016/j.amsu.2020.06.017

**Published:** 2020-06-18

**Authors:** Obada Hasan, Mashal Amin, Umar Rabbani, Amna Rabbani, Fatima Mahmood, Shahryar Noordin

**Affiliations:** a Orthopaedic & Rehabilitation Department, University of Iowa, United States; b Department of Pediatric Medicine, The Aga Khan University Hospital, Pakistan; c The Aga Khan University, Pakistan; d Department of Surgery, Section of Orthopedics, The Aga Khan University Hospital, Pakistan

**Keywords:** January effect, July effect, Resident, Trainee, Hip fracture, Surgery, Complication

## Abstract

**Introduction:**

Induction of new residents and surgical trainees in most institutes occurs once a year. Fresh residents with no experience, may pose a threat to the surgical procedure outcome and there can be a potential increase in patients' morbidity and mortality as a result of this turnover. Literature is inconclusive about this effect. Our aim was to study the “new residents’ induction effect” on postoperative complications after hip fracture surgeries.

**Methodology:**

This is non funded non commercialized study from a university hospital. Investigators studied a retrospective cohort of 1045 adult hip fracture patients who were operated at our tertiary care and level 1 trauma centre of a metropolitan city between 2008 and 2018. We defined primary exposure as the time period of new resident's induction (January–March) with the primary outcome in-hospital and 30days postoperative complications. Cox proportional hazard algorithm analysis was done at univariate and multivariable levels reporting Crude Relative Risk (RR) and Adjusted Relative Risk (aRR), respectively. Results were reported in line with STROBE criteria.

**Results:**

There were 274 (26%) patients in exposed group out of whom 109 (40%) developed postoperative complications. Interestingly, patients who had their surgeries during the induction period of new residents had 8% less risk of developing postoperative complications. However, result was statistically insignificant at both univariate and multivariable levels with RR; 95% C.I of 0.9 (0.78–1.22) and aRR; 95% C.I of 0.9 (0.78–1.22) after adjusting for the all other independent variables.

**Conclusion:**

The association of new residents' induction on postoperative hip fracture surgery complications, although protective, was insignificant after controlling for the potential confounding effect of patients’ background and demographic characteristics. We recommend further multi-centre high powered studies to analyze this.

## Highlights

• Surgical management of hip fractures is the gold standard and most effective management option.
• Fresh residents with no experience, may pose a threat to the surgical procedure outcome.
• We studied “new residents' induction effect” on postoperative complications after hip fracture surgeries.
• This turnover's higher risk was statistically insignificant in the current study.

## 1 Introduction

Each year in July, there is induction of fresh, inexperienced medical graduates into residency programs in North America as the senior, more experienced residents graduate to take responsibilities as fellows and attendings. This ‘July effect’ refers to the potential increase in patient mortality, postoperative complications and medical errors as a result of this turnover [1,2]. This apprehension is shared by both patients and doctors and has led to extensive study and research with variable results. Englesbe et al. reported a higher risk of postoperative morbidity and mortality in patients who underwent surgeries during the months of July and August [3]. Similarly, another study concluded that there is an increased rate of undesirable events at the beginning of academic year suggesting a lack of teamwork and communication amongst the many factors contributing to this increase [4]. Other authors, both in medicine and surgery, have failed to demonstrate a July effect [[5], [6], [7], [8], [9]].

A significant number of these studies have been conducted in western countries. There is scant data to suggest or refute the July effect in South Asian low middle-income countries. A recent study on a South Asian population found no evidence to support the hypothesis of new resident induction on increased patient morbidity and mortality [10]. Reports on the seasonal postsurgical outcomes of hip fixation surgeries in a South Asian population inspired us to analyze any correlation between resident inexperience and medical errors including perioperative and postoperative complications. It is important to mention that in contrast with the Western countries, the start of a new calendar year correlates with the influx of new residents at our institution and hence, we hypothesized to find an increase in postoperative complications during the months of January to March or a ‘January’ effect.

## 2 Methodology

### 2.1 Patients’ selection, study setting and eligibility

We retrospectively collected data of patients who were admitted from January 2008 to December 2018 at one of major tertiary care hospitals and level 1 trauma center in the country and region. We obtained approval from the University Ethical Review Committee (4543-Sur-ERC-16) and study was registered at clinicaltrials.gov. All adult patients (18 years and above), regardless of their gender and comorbidities, who had hip fractures that were managed surgically were included in the study. Patients with incomplete data or missing information in either the primary exposure or the outcome of interest were excluded. Moreover, we excluded patients with pathological fracture, open fractures, poly-trauma or revision surgeries. A total of 1045 patients were eligible for final analysis.

All orthopedic surgical procedures were performed by different orthopedic attendings. All patients received a standardized antibiotic prophylaxis protocol that was started an hour before surgery and continued for the next 24 h. Surgical site was prepped with pyodine soap/solution before draping. All patients underwent a standard postoperative protocol in the inpatient ward including mobilization and physiotherapy based on the procedure performed and the index surgeon's recommendations. This was followed up by home physiotherapy sessions on as need basis. Patients were closely followed up after discharge for 30 days to monitor for any postoperative complications that might have developed.

### 2.2 Data management, primary exposure, outcome and analysis plan

We recorded demographic data (age, gender, body mass index, comorbidities, American Society of Anesthesiologists grade, date of surgery, mechanism of injury, walking status prior to surgery and Charlson Comorbidity Index) and clinical data (type of procedure, duration of surgery, postoperative intensive care unit stay, length of hospital stay and in-hospital and 30 days postoperative complications).

Primary exposure was if patient was operated during the first three months of the year (January–March) when new enrollment of residents takes place. Month of surgery was extracted from the confirmed dates in our system from same source for all patients to reduce the possible information bias and was appropriately set as a categorical variable with 1, January; 2, February; 3, March and so on. We then decided to divide the months into 2 different time periods, one of three months, induction period (January–March) and the other 9 months as non-induction period (April–December). Primary Outcome of interest was postoperative complications (in-hospital and 30 days postoperative).

For descriptive purpose, frequency and percentages were reported for categorical variables (body mass index, American Society of Anesthesiologists grade, and Charlson Comorbidity Index); mean and standard deviation were reported for continuous variables (age and duration of surgery). To check associations, two groups of patients were compared for all their characteristics using *t*-test for age and duration of surgery and Pearson Chi-square test for BMI, ASA and CCI. Cox proportional hazard regression analysis was done reporting crude relative risk (RR) and adjusted RR (aRR) with 95% confidence interval (C.I.). For univariate analysis, *p* value of less than and equal to 0.25 was considered significant and for all multivariable analysis *p* value of less than and equal to 0.05 was considered significant. All plausible interactions were assessed. All statistical Analysis was done using STATA version 15. We reported the results based on STROCSS criteria [17].

## 3 Results

### 3.1 Description and demographic characteristics

**Fig. 1:**
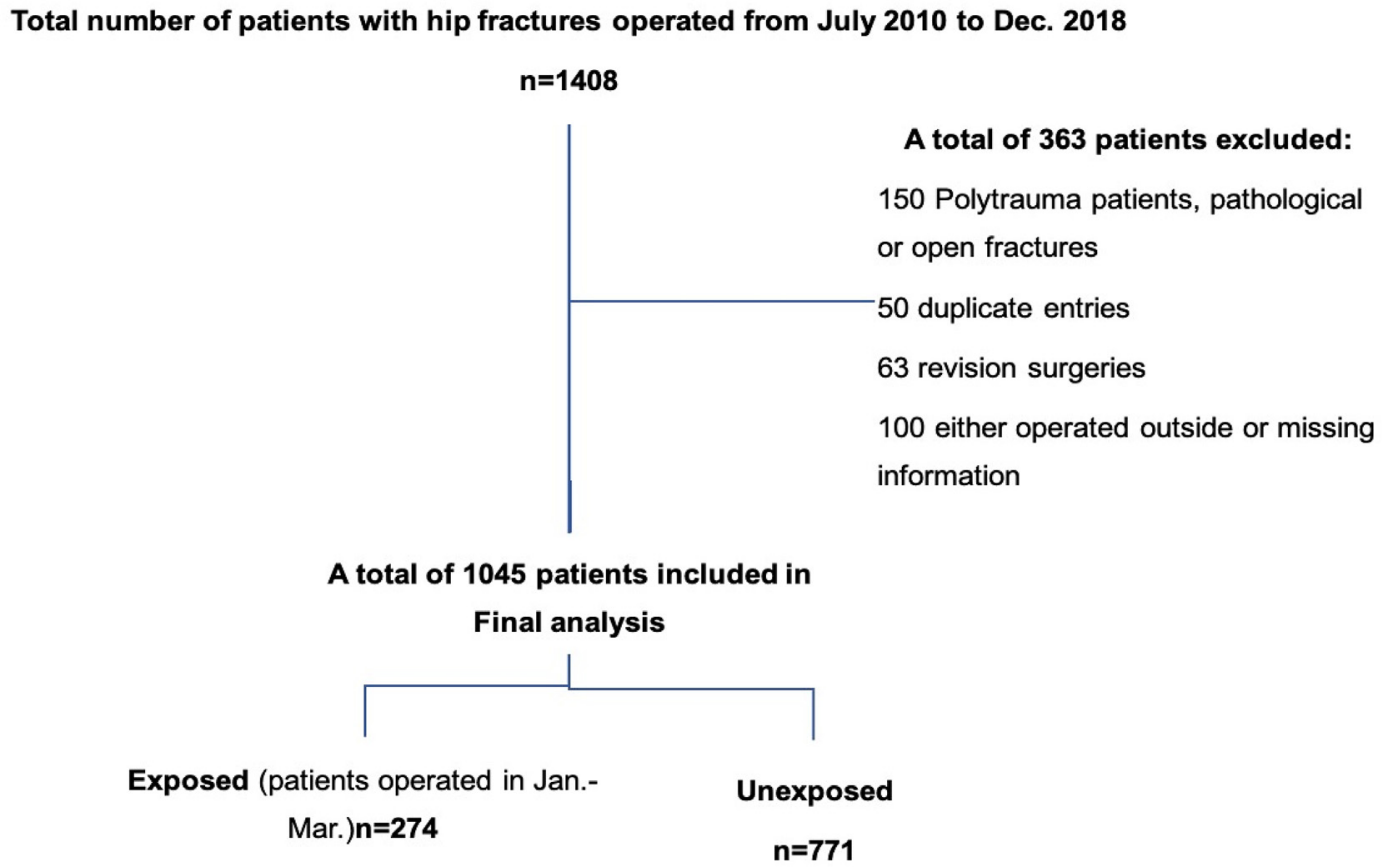
Study flow diagram.

After initial screening, a total of 1045 patients who underwent orthopedic surgeries for their hip fractures were included in the final analysis. Of these, 274 (26.2%) patients were exposed to their surgical procedure during the induction period of residents (January–March) and 771 (73.8%) patients underwent surgery during the non-induction period (April–December). Out of the total 274 patients in exposed group, 109 (40%) patients had post-operative complications while out of 771, 309 patients (42%) of the unexposed group had complications. However, result was statistically insignificant with RR; 95% C.I of 0.9 (0.78–1.22) (Fig. 1).

Amongst the group of patients who underwent their hip fracture surgery during the induction period of residents, the mean age was 68.7 ± 15 years, whereas; mean age of patients who underwent surgery during the non-induction period was 66.6 ± 15years. Mean duration of surgery in the induction period was lower than the mean duration of surgery in the non-induction period, i.e. 98 ± 42 and 104 ± 44 min respectively. During the induction period, 122 (45%) men and 152 (55%) of women had their surgery. Body mass index of the patients who had surgeries during the induction period and non-induction period were, 28 (10%) and 45 (5.0%) were underweight, 99 (36%) and 308 (40%) were normal weight, 111 (41%) and 314 (41%) were overweight, 36 (13%) and 104 (14%) were obese respectively. Proportions of patients who were admitted in ICU after surgery were same for induction period and non-induction period that is 10 (4.0%) and 38 (4.0%) respectively (Table 1).

**Table 1:** Demographic and clinical characteristics of exposed and unexposed groups.

Variables	Exposed n = 274	Unexposed n = 771	Variables	Exposed n = 274	Unexposed n = 771
**Age (Years)**	68.7 ± 15	66.6 ± 15	**Surgery duration**^a^**(Minutes)**	98 ± 42	104 ± 44
	**n(%) n(%)**			**n(%) n(%)**	
**Gender**^a^			**Anesthesia Type**^a^		
men	122(45%)	338(44%)	General	180 (66%)	579 (76%)
women	152(55%)	433(56%)	Regional	94 (34%)	192 (24%)
**BMI**			**ASA Status**^a^		
Underweight	28 (10%)	45 (5%)	I	14 (5%)	72 (9%)
Normal	99 (36%)	308 (40%)	II	93 (34%)	338 (44%)
Overweight	111 (41%	314 (41%)	III	143 (52%)	323 (42%)
Obese	36 (13%)	104 (14%)	IV	24 (9%)	38 (5%)
**CCI**			**Walking Status**		
NORMAL	19 (7%)	59 (8%)	Unknown	10 (4%)	22 (3%)
MILD	21 (8%)	85 (10%)	Independent	74 (27%)	226 (29%)
MODERATE	69 (25%)	228 (30%)	Limited community	74 (27%)	258 (33%)
SEVERE	165(60%)	399 (52%)	Walk with support	47 (17%)	93 (12%)
**Mechanism of Injury**			Limited home	69 (25%)	172 (22%)
Ground level fall	236 (86%)	645 (84%)	**Procedure**		
Others	38 (14%)	126 (16%)	DHS	143 (52%)	378 (49%)
**Fracture Type**			Bipolar Hemi.	27 (10%)	69 (9%)
IT	140 (51%)	383 (50%)	Monopolar Hemi.	44 (16%)	92 (12%)
NOF	115 (42%)	326 (42%)	THR	28 (10%)	95 (12%)
Sub trochanteric	19 (7%)	62 (8%)	IMN	10 (4%)	51 (7%)
			Others	22 (8%)	86 (11%)

a Significant with *p* value of ≤0.05. Proportions in the two groups are compared using Chi-square test and Wald χ2 test from Cox proportional algorithm model, while means±SDs and their *p* value by independent *t*-test.

### 3.2 Univariate analysis

On univariate level using the cox proportional hazard analysis, we observed that the induction of new residents had a post-operative complication risk of 0.9 with 95% CI (0.78–1.22). Age, BMI, ASA score, ICU admission, type of procedure, walking status, mechanism of injury and CCI were the significant variables with a *p* value of less than and equal to 0.25. Our results showed that with each year increase in the age of the patient, the risk of post-operative complications increased by 1.01 times [RR = 1.01, CI = (1.00–1.02)]. Patients with ASA level 4 had a risk of having post-operative complication 2.5 times higher as compared to a patient with ASA level 1 [RR = 2.51, CI = (1.46–4.32)]. Patients who were obese had a risk of developing post-operative complication 1.36 times higher as compare to patient with normal BMI [RR = 1.36, CI = (1.02–1.81). Patients who were admitted to ICU had a risk of developing a post-operative complication 1.8 times higher as compare to patients who were not admitted in the ICU [RR = 1.81, CI = (1.24–2.64)]. Patients with severe comorbid in CCI were found to have 2.1 times higher risk of developing a post-operative complication as compared to a normal CCI [RR = 2.09, CI = (1.31–3.33)] (Table 2).

**Table 2:** *Predictors of post-operative complications studied at univariate level using Cox regression method* reporting RR with 95% Confidence Interval.

Variables	RR (95% CI)	*p* value	Variables	RR (95% CI)	*p value*
**Residents' Induction Period**		0.85	**Fracture Type**		0.84
(Jan.–Mar.)	0.9 (0.78–1.22)		IT (ref)	1	
**Age**	1.01 (1.00–1.02)	<0.01	NOF	1.04 (0.85–1.27)	
**Surgery Duration**	1.00 (0.99–1.00)	0.82	Subtrochanteric	1.09 (0.75–1.56)	
**Gender**		0.26	**Type of Procedure**		0.09
Men (Ref)	1		Elective (ref)	1	
Women	1.11 (0.92–1.35)		Emergency	0.84 (0.69–1.02)	
**BMI**		0.16	**Walking Status**		0.06
Normal (ref)	1		Independent (ref)	1	
Underweight	0.91 (0.60–1.38)		Unknown	1.01 (0.54–1.88)	
Overweight	1.04 (0.83–1.29)		Limited Community Ambulant	1.42 (1.10–1.83)	
Obese	1.36 (1.02–1.81)		Walk with Support	1.36 (0.99–1.87)	
**ASA level**		<0.01	Limited Home Ambulant	1.20 (0.90–1.59)	
I (ref)			**CCI**		<0.01
II	1.56 (1.00–2.44)		Normal (ref)	1	
III	1.92 (1.23–2.98)		Mild	1.28 (0.72–2.26)	
IV	2.51 (1.46–4.32)		Moderate	1.76 (1.08–2.86)	
**ICU Admission**		<0.01	Severe	2.09 (1.31–3.33)	
No (ref.)	1		**Mechanism**		0.01
Yes	1.81 (1.24–2.64)		Ground level fall (ref)	1	
			Others	0.69 (0.52–0.94)	

Abbreviations: Jan.–Mar. = January to March Ref. = Reference Category; CCI= Charlson Comorbidity Index; Mech. = Mechanism of Injury; IT=Intertrochanteric; NOF= Neck Of Femur.

### 3.3 Multivariable analysis

Final model after a stepwise approach in multivariable analysis included the primary exposure “induction period” and three independent variables, age, ASA and ICU admission. Patients who had their hip fracture surgeries during the induction period of residents (January–March) had 8% less risk of developing post-operative complications as compared to patients who underwent surgeries during the other months (April–December) [aRR = 0.92, CI = (0.74–1.15)] after adjusting for all other independent variables. Patients who had an advanced ASA levels as II,III and IV had a higher risk of developing a post-operative complication as compared to patients with ASA level 1, after adjusting for all other variables [aRR = 1.29, CI (0.81–2.05)], [aRR = 1.46, CI = (0.91–2.33)] and [aRR = 1.83, CI = (1.03–3.25)] respectively. Patients who were admitted in the ICU after surgery had a 1.6 times higher risk of developing postoperative complications as compared to patients who were not admitted to the ICU, after adjusting for all other variables [aRR = 1.61, CI (1.10–2.36)] (Table 3).

**Table 3:** **Final model after multivariable analysis for factors associated with postoperative complications after hip fracture surgery**.

VARIABLE	aRR^a^ (95% CI)	p Value
Induction Period (Jan.–Mar.)	0.92 (0.74–1.15)	0.48
Age	1.01 (1.00–1.02)	0.003
ASA		(<0.01)
II	1.29 (0.81–2.05)	
III	1.46 (0.91–2.33)	
IV	1.83 (1.03–3.25)	
ICU Admission	1.61 (1.10–2.36)	0.01

a aRR = Adjusted Relative Risk (95% Confidence Interval); Jan.–Mar. = January to March.

## 4 Discussion

The results of our study did not support the hypothesis of a January effect in our tertiary care hospital located in a low middle income South Asian country. In other countries, it's called "The July Effect" which is the hypothetical increase in patient morbidity and mortality during the month of July when newly graduated doctors take on new roles as residents. The induction of residents at our University Hospital occurs in January.

There was no significant difference in the number of patients who developed post-operative complications in the induction period (40%) as compared to the ones operated upon during the rest of the year (42%). This is similar to the findings of Bohl et al. who studied the July effect in primary total knee and hip arthroplasty and found that there was no significant increase in serious adverse events (SAEs) during the first academic quarter versus the rest of the year (3.9% versus 3.6%, OR = 1.1, 95% CI = 0.7–1.5, P = 0.760) [5]. However, he did find an increase in the rates of any adverse events (AAEs) in the months of July to September. However, this seasonal variation in the increase in adverse events was also present in cases without resident involvement, so he attributed the increase to some other seasonal variants. In our study, we found that ASA level, obesity, severity of CCI and post-surgery ICU admissions were directly related to an increase in postoperative complications. We, however, did not categorize 30-day postoperative complications into AAEs and SAEs.

Mitchell et al. did a study on seasonal variation and post-operative outcomes specifically focusing on the 20 complications listed in the ACS-NSQIP [11]. He reported a statistical difference in19 of the 20 complications when compared to different quarters. The only complication with a statistically significant increase during the months of July to December was transfusion. However, this remained unexplained in his study as he concluded that there was no statistically significant increase in 30-day complications throughout the year, refuting the July effect.

Very limited data is available on the effect of July phenomenon in hip fracture repair surgeries. We therefore reviewed other orthopedic specialties to look for a relationship between resident involvement and an increase in patient morbidity. Rao et al. studied the July effect in Total Shoulder Arthroplasty and found no significant difference in postoperative complications in surgeries performed from July to September compared to the rest of the year(8). In addition, he specifically found no relationship between resident involvement and post-operative complications, and attributed several other factors including elevated temperatures and humidity to the increase in adverse events while not finding the existence of July effect in TSA.

Our results are in concordance with an analysis done by Cvetanovich et al. on total shoulder arthroplasty and July effect who concluded that resident involvement has no effect on 30-day postsurgical complications [12]. Similarly Haughom et al. in a large scale comprehensive analysis of 24,529 total knee arthroplasty cases also concluded that resident involvement was not a risk factor for short term patient mortality and morbidity, as no increase in complications was noticed with resident involvement [13]. He reported similar findings in another study on Total Hip Arthroplasty and resident involvement, further strengthening the findings of his previous study [14].

Bagsby et al. conducted a retrospective study on supracondylar humerus fractures and failed to demonstrate a July effect [15]. He categorized the data into early (July and August) and late (may and June) groups. Similar to our study, there was no significant increase in complications (7.0% (early) vs. 2.1% (late), *p* value 0.06) or operative time (33.32 ± 24.74 (early) vs. 28.63 ± 10.06 (late) min, *p* value 0.07) in the early vs late group. Surprisingly, in our study, the mean duration of surgery was lower in induction period compared to the non-induction period (98 vs 104 min with *p* value of 0.01).This could be attributed to an increase in the intraoperative attending oversight and lesser active involvement of the newly inducted inexperienced residents. In contrast to that, increased hands-on involvement of resident as the months pass by could be one of the contributing factors towards the increased operative times in the non-induction period.

However, not all data refutes the existence of a July Effect, both in orthopaedic literature and Internal Medicine. A multi-institutional cohort study done by Englesbe et al. found that there was a higher risk of patient morbidity and mortality during the months of July and August [3]. However, he could not specifically attribute the increase in patient mortality to resident inexperience as the study only included academic centers and there was no control group of any hospital without a training program. In addition to that, the patient mortality was almost as high in the month of December as in June which is in contradiction with the hypothesis of July effect, reflecting that there are other factors that potentially contribute to seasonal variation in patient outcomes.

Anderson et al. conducted a study on 324,988 patients with femoral neck and intertrochanteric fractures and reported that although there was a significant increase in patient mortality, intraoperative complications and length of stay in July/August in teaching hospitals but per se did not demonstrate a July Effect [16].

Our study has several caveats. We performed the study retrospectively and had a small sample size. This increases the risk of type 2 error. There is the need for a multi-center trial in a South Asian population which can help eliminate the bias. Nevertheless, large cohort studies involving multiple institutions have been done in the Western countries and support our findings. Furthermore, we did not have the data on the level of individual resident training and experience involved in the surgeries. Similarly, we did not know the experience of the whole surgical team ranging from anesthesiologists to attendings to the operating room technicians. Another factor which may have an impact on our results is the index of difficulty of each case. Although we did control for the most common variables including patient age, gender, BMI, comorbids and ASA levels, to name a few, the measurement of individual resident and operating room team experience is difficult to measure in a retrospective study.

## 5 Conclusion

The association of new residents' induction on postoperative hip fracture surgery complications, although protective, was insignificant after controlling for the potential confounding effect of patients’ background and demographic characteristics. We recommend further multi-center high powered studies to analyze this.

## Disclaimer

None.

## Financial support and sponsorship

Nil.

## Declaration of competing interestCOI

There are no conflicts of interest.

## Provenance and peer review

Not commissioned, externally peer reviewed.
